# DRAQ5 and Eosin (‘D&E’) as an Analog to Hematoxylin and Eosin for Rapid Fluorescence Histology of Fresh Tissues

**DOI:** 10.1371/journal.pone.0165530

**Published:** 2016-10-27

**Authors:** Katherine N. Elfer, Andrew B. Sholl, Mei Wang, David B. Tulman, Sree H. Mandava, Benjamin R. Lee, J. Quincy Brown

**Affiliations:** 1 Dept. of Biomedical Engineering, Tulane University, New Orleans, Louisiana, United States of America; 2 Dept. of Pathology and Laboratory Medicine, Tulane University School of Medicine, New Orleans, Louisiana, United States of America; 3 Dept. of Urology, Tulane University School of Medicine, New Orleans, Louisiana, United States of America; Pennsylvania State Hershey College of Medicine, UNITED STATES

## Abstract

Real-time on-site histopathology review of biopsy tissues at the point-of-procedure has great potential for significant clinical value and improved patient care. For instance, on-site review can aid in rapid screening of diagnostic biopsies to reduce false-negative results, or in quantitative assessment of biospecimen quality to increase the efficacy of downstream laboratory and histopathology analysis. However, the only currently available rapid pathology method, frozen section analysis (FSA), is too time- and labor-intensive for use in screening large quantities of biopsy tissues and is too destructive for maximum tissue conservation in multiple small needle core biopsies. In this work we demonstrate the spectrally-compatible combination of the nuclear stain DRAQ5 and the anionic counterstain eosin as a dual-component fluorescent staining analog to hematoxylin and eosin intended for use on fresh, unsectioned tissues. Combined with optical sectioning fluorescence microscopy and pseudo-coloring algorithms, DRAQ5 and eosin (“D&E”) enables very fast, non-destructive psuedohistological imaging of tissues at the point-of-acquisition with minimal tissue handling and processing. D&E was validated against H&E on a one-to-one basis on formalin-fixed paraffin-embedded and frozen section tissues of various human organs using standard epi-fluorescence microscopy, demonstrating high fidelity of the staining mechanism as an H&E analog. The method was then applied to fresh, whole 18G renal needle core biopsies and large needle core prostate biospecimen biopsies using fluorescence structured illumination optical sectioning microscopy. We demonstrate the ability to obtain high-resolution histology-like images of unsectioned, fresh tissues similar to subsequent H&E staining of the tissue. The application of D&E does not interfere with subsequent standard-of-care H&E staining and imaging, preserving the integrity of the tissue for thorough downstream analysis. These results indicate that this dual-stain pseudocoloring method could provide a real-time histology-like image at the time of acquisition and valuable objective tissue analysis for the clinician at the time of service.

## Introduction

Real-time assessment of freshly removed, intact tissue specimens can help improve clinical procedures, such as diagnostic biopsy, collection of samples for genetic or molecular testing, and/or surgical tumor resection. The current approach of frozen section analysis (FSA) for in-procedure histopathology is relatively time-consuming and damaging to the tissue and is not feasible for the immediate evaluation of small needle core biopsies [1]. Touch preparation of these specimens is currently the most effective method, but can frequently misrepresent tumor content and often needs a specialized cytopathologist for proper interpretation [2]. The labor-intensiveness and destructiveness of traditional histopathology preparations, like formalin-fixed, paraffin-embedded processing or frozen section analysis, is due to the need to deposit a thin section of hematoxylin and eosin (H&E) stained tissue on a slide to enable it to be evaluated by light-transmission microscopy. Emerging advanced microscopy techniques promise to eliminate the cutting step, enabling non-destructive imaging of the fresh, fully-intact biopsy. In particular, *ex vivo* microscopy methods that leverage fluorescent stains, such as confocal microscopy [3–10] or fluorescence structured illumination microscopy [11–14], are a direct analog to traditional pathology in terms of image contrast, enabling accurate assessments by trained pathologists. An alternative to FSA or touch-prep could be achieved by the application of topical fluorescent stains onto fresh, unprocessed and unsectioned tissues, which are able to mimic the pathological features of traditional hematoxylin and eosin (H&E) staining [15, 16]. Topical fluorescent staining is quick [14, 17], on the order of seconds, and when paired with a suitably rapid *ex vivo* microscopy system, could allow virtual H&E images of fresh tissue surfaces to be obtained within minutes of removal from the patient. This type of procedure, which also conserves the tissue for cryopreservation, could represent an attractive processing alternative to FSA and/or touch preparation for in-procedure pathology and triage of small biopsy samples for downstream analysis.

In order to reproduce the effects of H&E stains, a dual-component image contrast method suitable for *ex vivo* microscopy is needed that highlights the same nuclear, cytoplasmic, and extracellular features as H&E. Previous work on dual component contrast for *ex vivo* microscopy has used acridine orange (AO) for nuclear contrast combined with either reflectance [5, 18] or eosin [19] as a counterstain to achieve a fluorescent replacement for H&E. Acridine orange (AO) is a cationic stain commonly referred to as a nuclear stain; however, in reality it binds DNA, cytoplasmic RNA, muscle fibers and other cellular structures such as adipocyte cell walls [11, 20–22]. This nonselective staining makes it useful as a general comprehensive fluorescence histology stain, as we and others have demonstrated [13, 14, 23], but it also limits its utility as a fluorescent H&E analog since it demonstrates a staining mechanism that mixes features of both hematoxylin and eosin. Also, since AO binds cytoplasmic RNA, it does not specifically label the nucleus like hematoxylin. The inability to specifically label the nucleus in cells rich in cytoplasmic RNA makes it difficult or impossible to discern useful pathologic features, such as nuclear spatial density, nuclear-to-cytoplasmic ratio, nuclear pleomorphism, and nucleomegaly, which are important features of neoplasia assessed on H&E pathology. DRAQ5, on the other hand, is a live-cell nuclear stain with excitation and emission properties in the far red to near infrared spectrum (λ_ex_ = 647 nm, λ_em_ = 665–780 nm) that binds DNA stoichiometrically and exclusively, and is commonly used as a marker for DNA content in flow cytometry [24, 25]. The nuclear specificity of DRAQ5 suggests its potential use as a hematoxylin replacement in a fluorescent H&E analog. In the standard H&E stain, eosin acts as an anionic counterstain to hematoxylin. Fortunately, eosin is yellow-fluorescent under blue-green excitation (λ_ex_ = 490nm, λ_em_ = 530- 620nm) and is therefore a spectrally compatible fluorescent counterstain to DRAQ5 [26].

In this work, we evaluated the use of DRAQ5 and eosin (“D&E”) as a direct fluorescent analog to traditional H&E staining on fresh, unsectioned (‘zero-cut’) tissues at the point-of-acquisition. The fidelity of D&E staining compared to traditional H&E was first validated in frozen and fixed sections from a variety of human tissues. The intended use of D&E for virtual histology of *ex vivo* uncut human tissues was then demonstrated on human kidney and prostate biopsies using structured illumination fluorescence microscopy, which is one method of obtaining thin optical sections of tissue. D&E for renal biopsy diagnosis was used to identify normal and sclerotic glomeruli, as well as clear cell renal cell carcinoma in simulated 18G needle-core biopsies without any tissue processing other than immersion in the dyes. D&E was also used in the assessment of prostate cancer biospecimen quality by evaluating the amount of prostate cancer present in a simulated large-core biopsy. The topical staining method introduced here, combined with fluorescent *ex vivo* microscopy, could enable routine non-destructive histological assessment of fresh human tissues at the point-of-care for a variety of applications.

## Materials and Methods

### Stains

DRAQ5 (5 mM, Biostatus, Ltd.) was diluted from 5 mM to 50 μM in PBS. Eosin Y (E4009, Sigma-Aldrich) was dissolved to [2% v/v] in 80% ethanol. Stains were applied directly to tissue sections and intact tissues without further modification.

### Tissue collection and processing

Formalin-fixed paraffin embedded (FFPE) tissue sections (n = 11) were obtained from the Tulane Medical School Histology core under an IRB-approved protocol. De-identified, large, paraffin-embedded tissues were selected as examples of unique tissue morphology of different organs. The tissues were cut into 4 μm thick sections and mounted on microscope slides. They were stored at room temperature until immediately prior to staining, at which point the sections were de-paraffinized, stained according to the protocol below and imaged with wide-field epifluorescence microscopy. After fluorescence imaging, they were processed for H&E through the Tulane Medical School Histology Department and scanned at 20X with an Aperio whole slide scanner (Leica Biosystems).

Frozen section tissues (n = 22) were obtained from the Louisiana Cancer Research Center (LCRC) Biospecimen Core under an IRB-approved protocol. Flash-frozen, de-identified tissue biopsies from various organs were processed into 10 μm thick sections then placed onto standard glass microscope slides. They were then stored frozen at -18°C until immediately prior to staining and imaging the tissue. The slides were removed from the freezer, gently rinsed with room temperature PBS to thaw the tissue, stained using the protocol described below, and imaged. After imaging, the sections were sent to Tulane Medical School Histology Department for standard H&E processing, then scanned with the whole slide scanner.

Fresh renal biopsies (n = 3) and prostate biopsies (n = 1) for this study were obtained in accordance with an Institutional Review Board-approved protocol. Biopsies were collected from excised tissue specimens for purposes of research and were not collected for clinical diagnosis, therefore we use the term “simulated biopsy” when referring to them. Simulated renal biopsies were obtained using an 18G core-needle biopsy device (Bard Monopty) from freshly excised (*ex vivo*) partial and total nephrectomy specimens from benign and adjacent neoplastic renal tissues. Prostate biopsies were taken as punches from radical prostatectomy specimens containing prostatic adenocarcinoma. Intact biopsies were stained and imaged according to the protocol below, then sent to the Tulane Medical School Histology Department for fixation, sectioning, and standard H&E processing. Resulting H&E sections were scanned with the previously described whole slide scanner.

All research was conducted under a protocol approved by the Tulane University Biomedical Institutional Review Board. Specimens were de-identified prior to acquisition and data were analyzed anonymously, therefore informed consent was not required.

### Thin tissue section staining and fluorescence imaging

A validation study was conducted on de-paraffinized FFPE and thawed frozen sections stained with D&E. A section of tissue was briefly exposed to DRAQ5 by immersing the section in 50 μM DRAQ5 for ten seconds. Then 2% eosin in ethanol was applied for 10 seconds and then thoroughly rinsed with PBS to remove any excess stain. The tissue section was then exposed to 50 μM of DRAQ5 again for three minutes before a single rinse with PBS. The double-exposure protocol for DRAQ5 was developed after an initial staining optimization demonstrated a marked increase in intensity of DRAQ5 compared to single exposures staining before or after eosin application. Eosin intensity was not significantly affected by subsequent application of DRAQ5. Samples were imaged with a custom epi-fluorescence microscope with optical sectioning structured illumination microscopy (SIM) capability based on an automated modular platform (RAMM, Applied Scientific Instrumentation) and described in detail in our previous publications [13, 14]. Briefly, the system consists of a 470 nm LED for eosin excitation (M470L2, Thorlabs), and was modified in this work to include a 630 nm LED for DRAQ5 excitation (UHP-Mic-LED-630, Prizmatix). The LEDs were combined with a dichroic beam combiner (Prizmatix) and imaged onto a ferroelectric spatial light modulator (SLM, 3DM, Forth Dimension Displays), which was used to project patterns for structured illumination microscopy onto the sample through a 10X, 0.45NA Plan Apo objective lens (Nikon) in epi-illumination configuration. A multiband dichroic beamsplitter (FF409/493/573/652-Di01-25x36, Semrock) and emission bandpass filter (FF01-432/515/595/730-25, Semrock) was used to allow excitation and emission of both DRAQ5 and eosin; the DRAQ5 and eosin images were taken sequentially at each frame position by illuminating with their respective LEDs. The sample was imaged through the 10X objective and a Nikon tube lens onto a Hamamatsu Orca Flash 4.0v2 scientific CMOS camera. The system was controlled through custom-written LabVIEW software. The single frame field of view for the system is 1.3 x 1.3 mm at 4.2 megapixel resolution (2048 x 2048 pixels). The lateral resolution for eosin emission is 1.47 μm and for DRAQ5 emission is 1.90 μm, both limited by the optical point spread function. Images of larger areas were taken by translating the sample over the objective and taking multiple frames, which were assembled into mosaics after imaging, similar to digital whole slide scanners. For tissue section imaging, the microscope was operated in standard wide-field illumination (non-SIM) epifluorescence mode. Both channels used an integration time of 110 ms and each LED intensity was adjusted to maximize signal while not saturating the camera.

### Intact, whole biopsy staining and fluorescence structured illumination microscopy

Adjustments were made to the staining protocol for whole pieces of tissue versus tissue sections on slides. Whole biopsies were submerged in PBS immediately after collection until time of staining. The biopsies were then submerged in [2% v/v] eosin for 10 seconds and rinsed by immersion in phosphate buffered saline (PBS) to remove excess stain. The biopsy was then blotted dry with lab tissue (Kimtech) and submerged in 50 μM DRAQ5 for 3 minutes. After incubation in DRAQ5, the biopsy was rinsed by immersion in PBS and dried by patting with lab tissue. The stained tissue was placed on a glass slide ensuring maximum contact with the surface of the slide. The entire staining process took approximately 5 minutes and could be parallelized for multiple biopsies using cassettes.

For fresh, uncut tissue imaging the microscope was operated in structured illumination microscopy mode using an absolute spatial frequency of the grid pattern at the sample of *f* = 17.1 cycles/mm and an integration time of 100 ms—110 ms, with the power of each LED adjusted to maximize signal without saturating the camera in wide-field mode. The normalized spatial pattern frequency (ν) for each excitation channel was determined using the relationship *v* = *f* λ_ex_ / NA, where λ_ex_ is the center excitation wavelength of the respective LED and NA is the numerical aperture of the objective lens [13]. Using ν and the center emission wavelengths of the eosin and DRAQ5 channels, theoretical calculations for the axial response of the system in terms of half-width-half-maximum (HWHM) of each channel was made following Karadaglic and Wilson [27]. The HWHM is used as it most closely approximates the thickness of tissue sampled at the planar surface in contact with the glass slide. At *f* = 17.1 cycles/mm, the theoretical HWHM of the eosin channel is 18.8 μm and the HWHM of the DRAQ5 channel is 18.7 μm. Our previous work demonstrated that the theoretical calculation closely aligns with the measured response of the system using the eosin (470 nm excitation) channel [13]. Although H&E section thicknesses vary between institutions, typical paraffin and frozen section cutting plane thickness at our institution can range from 4 μm to 10 μm. Similar to a confocal microscope, the optical section thickness of the structured illumination microscope may be adjusted to get section thicknesses approximating those of H&E sections, in this case by changing the grid pattern frequency to higher values to achieve thinner optical sections. However, this comes at the expense of decreased signal recovery and decreased SNR, and we have found that the frequencies used in this work represent an acceptable tradeoff between SNR and increased contrast due to background rejection with optical sectioning.

Using structured illumination microscopy mode it took 330 ms to image an area of 1.3 mm x 1.3 mm per channel for the stains used in this work. A typical 18G biopsy tissue area of 33.8 mm^2^ can be stained using the above protocol within 5 minutes, placed on a slide, and imaged in slightly greater than 10 seconds per channel, allowing for motorized stage (7mm/sec lateral translation speed) adjustment. Multiple biopsies may be placed in a cassette and stained concurrently, such that increasing the number of biopsies to be imaged increases the total processing time only by the imaging time per biopsy and not the staining time. By increasing illumination intensity to improve signal, imaging time could be further reduced by a factor of 10 to 30 ms per frame as shown in our prior work using a very bright fluorophore, acridine orange [13, 28]. After imaging, the imaged surface of the biopsy was marked with histological ink and fixed in 10% formalin for a minimum of 48 hours. The biopsies were sent to the Tulane Histology Department for standard H&E processing where a 4 μm section was cut from the biopsy, enabling comparison of the H&E section against the D&E pseudocolored image of the intact biopsy.

### Digital pseudo-H&E staining of fluorescent images

Raw 16-bit grayscale TIFF images (either widefield images for tissue sections or SIM images for thick tissues using the RMS optical sectioning algorithm [14, 29]) were first converted to double precision arrays spanning the range [0 1] for further mathematical processing. In order to account for illumination uniformity, a flat-field correction procedure was performed by dividing each raw image frame by a calibration image of a uniform fluorescent sample for each respective channel, followed by construction of a mosaic from the assembled frames. The mosaicked image for each channel was further preprocessed using the following operations:

$$D5=A{I_{D5}}^{\gamma_{D5}}$$ (1)

$$E=B{I_{E}}^{\gamma_{E}}$$ (2)

where *I*_*D5*_ and *I*_*E*_ are the intensity corrected mosaics for each channel, *y*_*Δ*_₅ and *y*_E_ are non-linear weighting factors applied to compress the image intensities to improve visualization of the large-dynamic-range images, and *A* and *B* are empirically determined scaling coefficients used to match the mean intensities of the two channels. For all images, the gamma factors ranged from 0.65–1. The average *y*_E_ was 0.75; the average *y*_*Δ*_₅ was 0.85. The scaling coefficients typically ranged from 0.8 to 5, where *A* ranged from 0.8–3 and *B* ranged from 1.5–5.

Following these preprocessing steps, using the procedure first outlined by Gareau [5] and later improved by Bini et al. [15], we re-mapped the pixel intensities of the DRAQ5 and eosin channels of the pre-processed mosaics into a composite RGB image that simulated the appearance of H&E staining:

R:[1−D5(1−0.24)−E(1−0.88)] (3)

G:[1−D5(1−0.21)−E(1−0.27)] (4)

B:[1−D5(1−0.62)−E(1−0.66)] (5)

The individual RGB arrays were then re-scaled into a single composite 16-bit RGB TIFF image. All processing was performed in MATLAB.

### Validation study of D&E against H&E on tissue sections

We examined the performance of D&E on FFPE sections and frozen sections in order to compare D&E’s effectiveness as a diagnostic tool against standard H&E processing before applying it to thicker tissues. Serial staining and imaging (D&E followed by H&E) of thin sections was useful in order to examine the exact morphology and staining correspondence between the two methods. In order to test D&E on fixed tissue, the pathologist and other researchers were blinded to the source organ of each tissue section. A previous critique for many previous *ex vivo* imaging methods is that a pathologist would need to undergo a training period to adjust to the images generated by the new imaging modality. This training period is especially important if the images could possibly convey false or misleading data created by imaging artifact or contrast mechanisms which do not recapitulate standard histological contrast, which may lead to low reviewer certainty and resulting errors in interpretation. By using as close an approximation of the histochemical behavior of H&E as possible in fluorescence microscopy, the need for training could be vastly reduced or theoretically eliminated. This validation study was conducted in order to determine if a pathologist, with no prior training in review of D&E images, could correctly identify and diagnose a variety of tissues using this contrast mechanism. The blinded pathologist evaluated first the D&E image of the section and then the digitally scanned H&E section to determine the organ of origin, tissue type, and noted any distinctive morphological features commonly used in diagnostic evaluation. In total, seven tissue types were identified across twelve sections: lung (n = 3), prostate (n = 2), thyroid (n = 2), liver (n = 2), kidney (n = 1), adrenal gland (n = 1), and colon (n = 1). A second study was performed to evaluate the effectiveness of D&E on previously frozen tissue sections. These frozen sections were selected from various organs that are likely to be subjected to in-procedure histological assessments (prostate, lung, colon, breast, bladder, kidney, and skin) and may be subjected to ancillary molecular techniques for further diagnostic workup. As with the fixed tissue study, each section was evaluated to determine the organ of origin, tissue type, and noted any distinctive morphological features commonly used in diagnostic evaluation. Those sections that were so degraded by frozen artifact as to impair recognition in both D&E and H&E were excluded from analysis. A total of 25 sections were evaluated: lung (n = 6), prostate (n = 6), colon (n = 4), kidney (n = 3), breast (n = 3), and bladder (n = 3).

### Evaluation of D&E for ‘zero-cut’ pathology on intact fresh biopsies

A pilot study was conducted to test the use of D&E on intact whole needle core renal biopsies and large prostate biopsies. Three 18G core needle renal biopsies obtained from the previously described kidney samples were stained with D&E and imaged using fluorescence structured illumination microscopy. A large core needle prostate biopsy from the previously described prostate samples was also stained with D&E and imaged using fluorescence structured illumination microscopy. The pathologist examined the ability to differentiate pathologically relevant features and identify any evidence of disease in the D&E thick tissue images and their corresponding H&E tissue sections.

## Results

**Fig 1:**
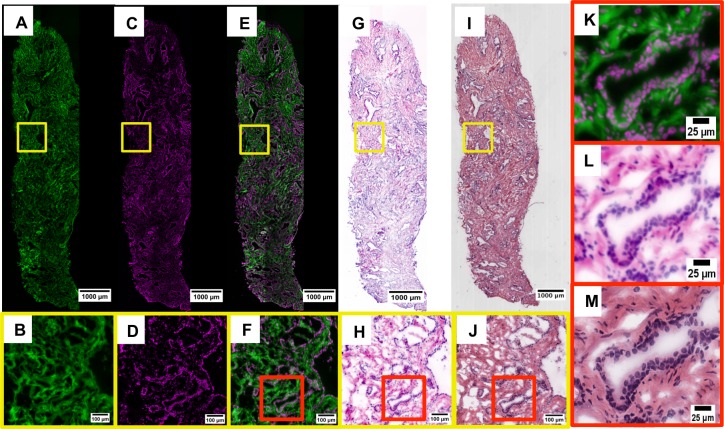
Fluorescent eosin channel (A, B). Fluorescent DRAQ5 channel (C, D). Fluorescent composite of D&E (E, F, K). Fluorescent D&E image pseudocolored to resemble H&E (G, H, L). The brightfield H&E of the corresponding histology (I, J, M).

To demonstrate the image collection and processing pipeline, Fig 1 shows a single 10 μm thawed frozen section of a prostate punch biopsy, stained with D&E, imaged, and recolored to simulate H&E, followed by standard H&E processing and imaging. This figure shows eosin staining all non-nuclear cellular material (Fig 1A and 1B), DRAQ5 bound to the nuclei (Fig 1C and 1D), and the combined image of the two channels in green/magenta pseudo-coloring (Fig 1E, 1F and 1K). The prostate section was later stained with H&E and then scanned with a digital slide scanner (Fig 1I, 1J and 1M). After re-coloring the D&E image to simulate the appearance of H&E (Fig 1G, 1H and 1L), the two images are shown to be highly similar. Specifically, in the eosin channel (Fig 1A and 1B), cytoplasmic and stromal material is clearly visualized as well as the absence of fluorescence signal where nuclei are located. The DRAQ5 channel (Fig 1C and 1D) demonstrates specific nuclear staining; the morphology of individual nuclei forming glands and fibroblast nuclei throughout the stroma are clearly visualized and spatially consistent with hematoxylin staining in H&E. Merging the two separate channels creates a single fluorescent image that shows nuclei, cytoplasmic and extracellular material together (Fig 1E, 1F and 1K). The fluorescent composite image of the DRAQ5 and eosin channels shows that the nuclear (magenta) and cytoplasmic/extracellular (green) compartments are each uniquely stained and spatially consistent with H&E. The individual fluorescent channels of A and C were then combined using Eqs 1–3 to create Fig 1G, 1H and 1L, recapitulating standard H&E histology (Fig 1I, 1J and 1M).

**Fig 2:**
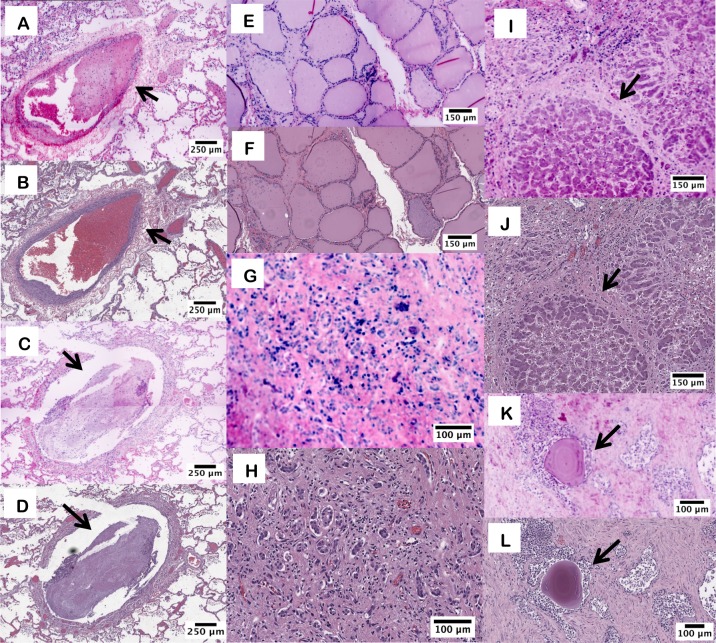
D&E and H&E images from FFPE tissue sections. D&E (A) and H&E (B) of lung parenchyma and small pulmonary artery branch with blood clot (arrow). D&E (C) and H&E (D) of bronchus with mucus plug (arrow). D&E (E) and H&E (F) of thyroid follicles. D&E (G) and H&E (H) of cirrhotic liver showing ductular reaction. D&E (I) and H&E (J) of liver with a cirrhotic nodule (arrow) and surrounding ductular reaction. D&E (K) and H&E (L) of prostate glands with corpora amylacea (arrow).

Fig 2 contains example images of FFPE sections from various tissue types imaged with both D&E and H&E, allowing a direct comparison of the tissue morphology and staining fidelity. The pathologist correctly identified the tissue type in all twelve D&E fixed sections in a blinded review. The comparison between methods demonstrated complete morphological correspondence. Specific regions of interest of particular tissues are shown in Fig 2. Unique morphological features in the same type of tissue, such as the distinction between a pulmonary artery (Fig 2A and 2B) and a bronchus (Fig 2C and 2D) were observed in both D&E and H&E versions of the tissue. Alveolar septae, bronchial epithelium, mucus, and blood clot show strong morphologic and color compatibility in both modalities. Thyroidal colloid and follicular cells [Fig 2E and 2F] are easily distinguished in the D&E and H&E images.

Although the pseudo-colored D&E replicates H&E with high fidelity spatially, potentially useful color variations between D&E-processed images and standard H&E are evident. Areas of liver cirrhosis were identified in both the D&E and H&E images (Fig 2G, 2H, 2I and 2J) and robust collagen fibrosis in the liver sections appears more strongly on the D&E sections. The D&E pseudocolor algorithm allows for increased contrast, as in the ductular reaction in liver tissue (Fig 2G and 2H). Additionally, the lamellations of the corpora amylacea are highly pronounced on the D&E as well (Fig 2K).

**Fig 3:**
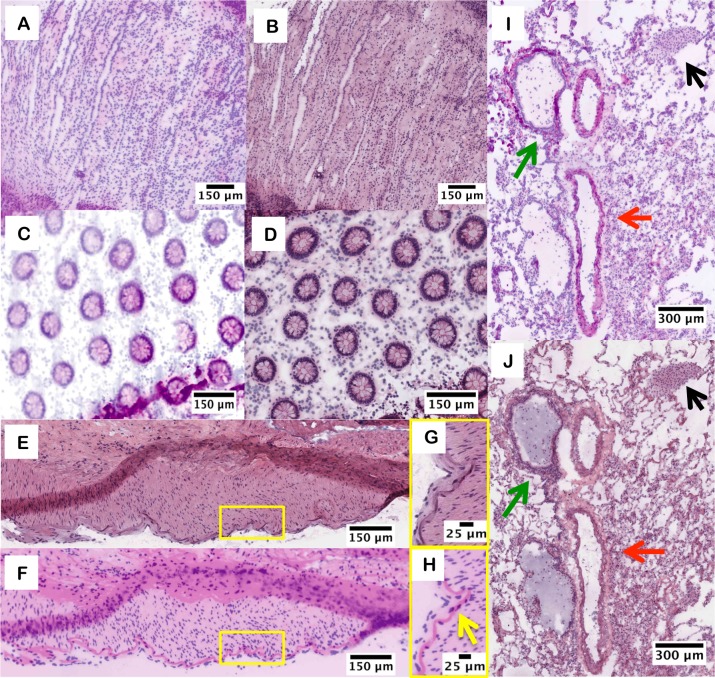
D&E and H&E images from frozen tissue sections. D&E (A) and H&E (B) of renal medullary tubules. D&E (C) and H&E (D) of colonic crypts. D&E (E, F) and H&E at (G, H) of outer pleural surface of lung with prominent elastic lamina (yellow arrow). D&E (I) and H&E (J) of lung showing pulmonary artery branch (red arrow) with terminal bronchiole (green arrow) and alveolar macrophages (black arrow).

The pathologist also correctly identified all twenty-five D&E frozen tissue sections. Regions of either morphological or pathological interest on frozen tissues were selected and are shown in Fig 3. Renal medullary tubules are seen (Fig 3A and 3B) as well as normal colonic crypts (Fig 3C and 3D). In a single area of lung tissue, identification of important morphological features including a pulmonary artery branch, terminal bronchiole and alveolar macrophages were all clearly identified on D&E and confirmed in H&E (Fig 3I and 3J).

Similar to the fixed tissue images, color variations are observed between D&E and H&E, although morphological (spatial) correspondence is preserved between methods. Some useful differences between the two staining methods exist, most notable of which is the emphasized elastic lamina seen in bright pink in the D&E version (Fig 3E and 3G) compared to the H&E version (Fig 3F and 3H) of the outer pleural surface of the lung. Typically, this structure is best visualized with a special elastin stain and is used to stage pleural invasion in lung carcinomas.

**Fig 4:**
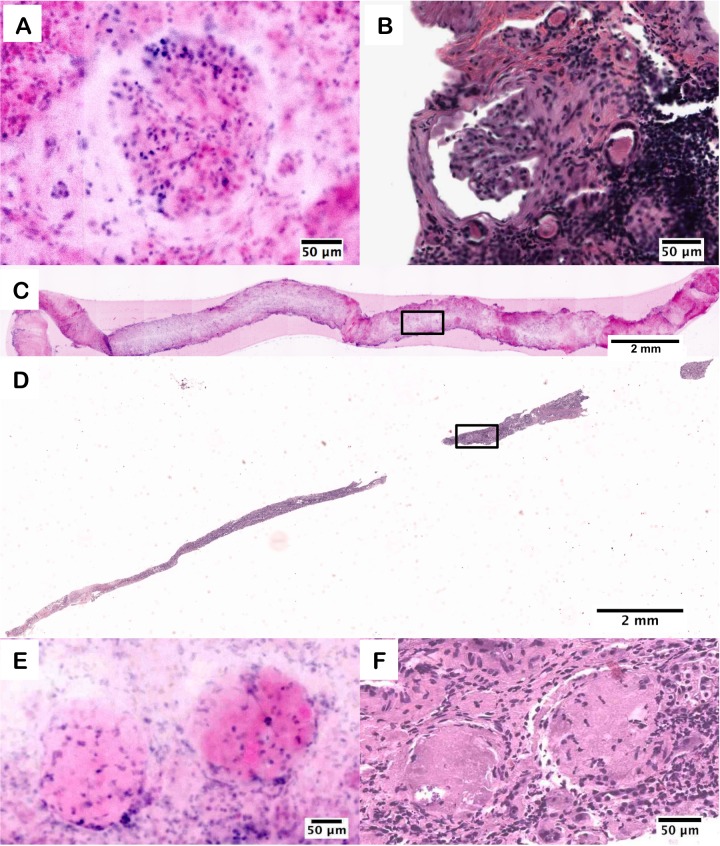
D&E (A) and H&E (B) of a healthy glomerulus from an intact kidney needle biopsy core. D&E (C) of an entire intact kidney needle biopsy core, and (D) subsequent H&E section. D&E (E) and H&E (F) of sclerotic glomeruli from the kidney biopsy.

The final and most important evaluation consisted of testing the capability for D&E to stain fresh, uncut tissues and still provide the same diagnostic information as H&E. Staining and imaging thick tissue is made more difficult by the need to control the stain penetration and background signal of the thick tissue, and it requires the use of a fluorescence microscope capable of creating an optical section in lieu of a physical section. Three intact, 18 G core needle simulated renal biopsies and one simulated prostate punch biopsy were fluorescently stained and imaged with structured illumination microscopy, which is an example of an emerging optical sectioning microscopy technique for *ex vivo* microscopy [11, 13, 14]. Pathologist review of the D&E images identified a healthy glomerulus in one biopsy and a sclerotic glomeruli pair, shown in Fig 4, which was confirmed in the H&E-stained FFPE section taken subsequent to imaging.

The D&E (Fig 4A) staining and imaging allowed the entire surface of the biopsy to be imaged *in toto*, compared to the fragmented appearance of the sectioned biopsy post-H&E processing (Fig 4B).

The area of the optical section in the biopsy D&E image is 20.5 mm^2^, whereas after physical sectioning and H&E processing, the remaining tissue section comprises an area of only 6.7 mm^2^. Multiple “virtual sections” of the biopsy can be collected without harm to the tissue using the D&E method, by physically rotating the biopsy on the microscope slide and repeating the imaging. Therefore, thorough coverage of the biopsy surface can be imaged with robust concordance between D&E and H&E analysis. Increased differences between the D&E and H&E images are apparent in the biopsy, but these can be attributed to the thickness of the tissue being imaged in D&E and the loss of tissue during standard histopathology processing. We were able to identify normal glomeruli in the first biopsy, as well as a sclerotic glomeruli pair in the second biopsy. The glomerulosclerosis seen in the second biopsy is characterized by a loss of nuclei and the replacement of the round glomerulus with eosinophilic fibrosis, seen in both the D&E and H&E images.

**Fig 5:**
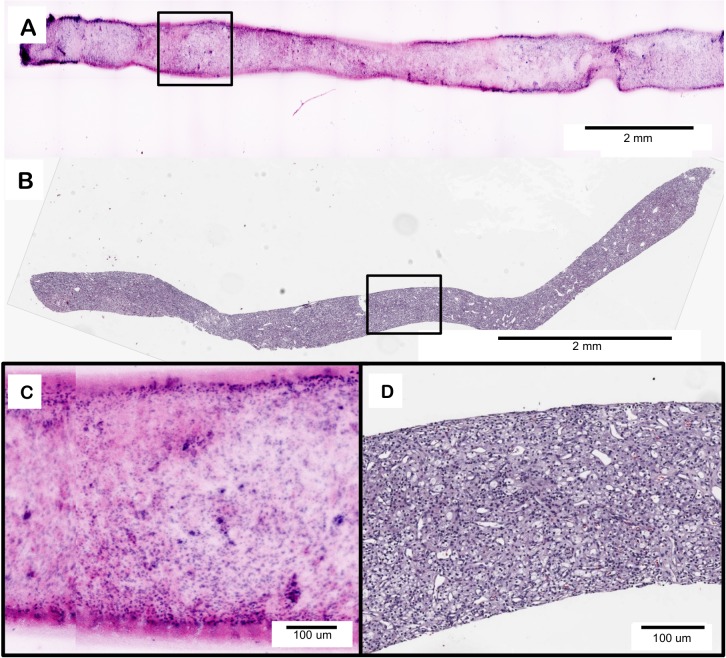
D&E (A) of clear cell renal cell carcinoma in an intact 18G core needle kidney biopsy and (C) an area of higher magnification showing loss of normal renal architecture. H&E (B) of a 4 *υ*m section from the same kidney biopsy showing clear cell renal cell carcinoma and (D) an area of an area of higher magnification showing loss of normal renal architecture.

Clear cell renal cell carcinoma (CCRCC) was diagnosed in an intact 18G renal core biopsy, shown in Fig 5. In both the D&E (Fig 5A) and H&E (Fig 5B) images there is a clear loss of normal renal architecture with replacement by a homogenous proliferation of neoplastic tumor cells, encompassing the entire biopsy specimen. The homogenous nature, lack of tubules and glomeruli, and light coloration of the eosin signal compared to the DRAQ5 signal (Fig 5C) are all features indicative of the presence of a neoplasm, confirmed as clear cell renal cell carcinoma on histology (Fig 5D).

**Fig 6:**
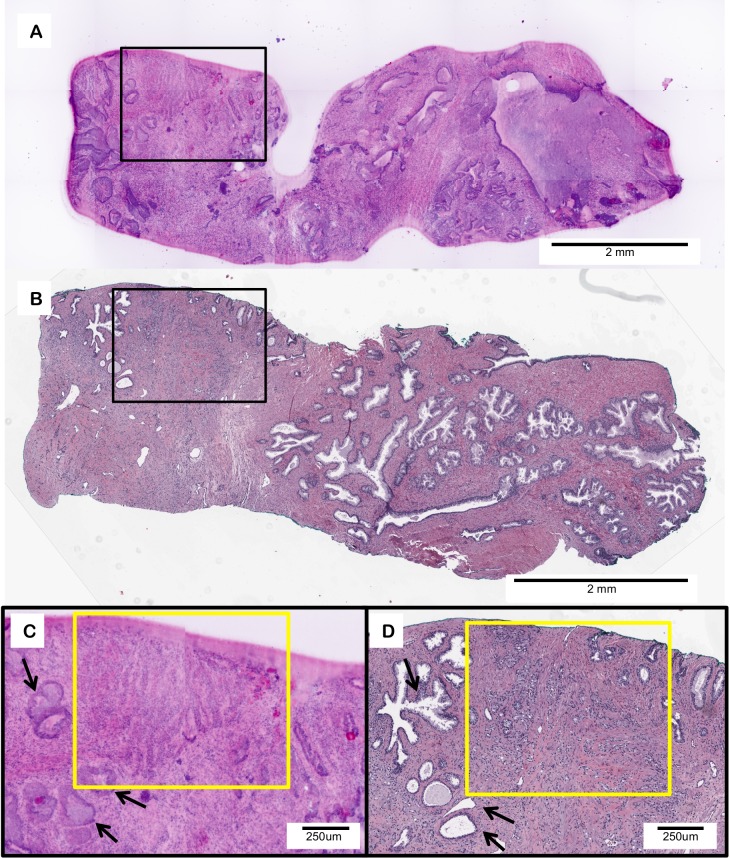
D&E (A) of an intact prostate large core needle biopsy with adenocarcinoma and a 4 *υ*m H&E (B) section from the same tissue. D&E (C) and H&E (D) of infiltrating prostatic adenocarcinoma (yellow box) adjacent to normal prostatic glands (black arrows).

Fig 6 provides an example of a simulated prostate biopsy containing adenocarcinoma. As an example of the use of D&E for assessment of cancer biospecimen quality, we evaluated the tumor content using each method; 10% tumor content was observed in the D&E image, matching the 10% tumor content observed in the H&E section. Within the biopsy, areas of healthy glandular structures (black arrows) are shown adjacent to areas of malignant adenocarcinoma gland infiltration (yellow box) in both D&E (Fig 6C) and H&E (Fig 6D) images. Tumoral content is comparable in both methods, supporting further the ability of D&E to be used as a non-destructive tissue triage method for personalized medicine and downstream molecular analysis.

## Discussion

In this report we demonstrate that the novel combination of fluorescent stains DRAQ5 and Eosin Y (D&E) allows for time-efficient staining and rapid fluorescence imaging to create virtual histologic images from fully-intact fresh core needle biopsy specimens. D&E staining was first validated in fixed and frozen tissue sections, and in all conditions robustly recapitulated the appearance of standard H&E histopathology. This validation study demonstrated the ability for a pathologist to correctly identify a variety of tissue types in both FFPE and frozen specimens with no prior training using this dual-stain fluorescent method. Importantly, pathologist review of the tissues found that diagnostically relevant morphological features were preserved between the methods. By applying the pseudocolor process described by Gareau [5] to D&E images, we were able to visualize the same morphological features as H&E. Both Figs 2 and 3 demonstrate the ability to identify histologically relevant features on D&E images, supported by the H&E images of the same sections. Importantly, the use of D&E does not damage or otherwise alter the tissue for future histopathology processing. Particularly significant is the demonstration of D&E as a direct means to create H&E-like images non-destructively from intact biopsy tissues, when combined with a form of fluorescence optical sectioning microscopy, as evidenced in Figs 4–6. One positive consequence of imaging the tissue in the fresh state as shown in Fig 4C and 4D is that the resulting optical sections are less susceptible to tissue fragmentation and oblique sectioning artifacts, potentially providing a more comprehensive assessment of the biopsy in a single image. We have also shown the ability to accurately and rapidly diagnose disease in thick, intact tissue without any cutting or additional processing, as shown with clear cell renal cell carcinoma in Fig 5 and prostate adenocarcinoma in Fig 6. Therefore, D&E can be paired with FSA and/or touch preparation for rapid on-site evaluation. Further studies examining the ability for pathologists to diagnose wide varieties of tissues and the interaction of D&E with downstream analyses may prove D&E with optical sectioning to be an alternative for FSA. The demonstration of rapid histologic imaging of fresh core biopsies could have immense clinical significance, as this would allow for point-of-care evaluation of core needle biopsies not only for diagnosis, but also for robust evaluation of tumor content, cellularity, and consequently DNA content for ancillary molecular testing. Additionally, the modality could also be utilized for medical renal biopsies with the evaluation of glomerular content for the triage of tissues to electron microscopy, histology, and immunofluorescence.

The method described here is a high fidelity analog to traditional H&E. The DRAQ5 channel may better isolate nuclear-only features compared to computational segmentation of nuclei from H&E images using color deconvolution or other methods (S1 Fig), supporting quantitative measurement of nuclear features with fewer required image processing steps (S2 Fig). Although D&E provides an extremely high fidelity match to H&E in terms of spatial correlation, we described a few variations between some non-nuclear structures in the D&E and H&E images, primarily due to the increased sensitivity to eosin concentration caused by fluorescence imaging rather than brightfield absorptive imaging. These variations were not considered to be detrimental to diagnosis, and in fact they contained useful features that with further characterization could provide advanced diagnostic abilities not typically available in H&E. For example, some structures with diagnostic relevance, such as the eosin-stained elastic lamina of lung pleura (Fig 2G and 2H) were enhanced in the D&E image compared to H&E. While matching the hue of the H&E image to the D&E image could render the elastic lamina of both indistinguishable, it is possible that the positive contrast and hue differences of D&E may allow for easier visualization of highly-stained features compared to the negative absorbance contrast of bright field microscopy. For staging non-small cell lung carcinoma, stains other than H&E are often needed in order to fully examine the anatomical structures of the elastic lamina. With increased invasion of the visceral pleura, these structures become increasingly difficult to identify [30]. The combined intense hue and intensity of D&E may allow for this examination without additional contrast agents; the H&E slides would need to be digitally enhanced to create artificial intensity and contrast in order to match the differentiation afforded by D&E.

The methods used to apply eosin for D&E staining are different from those used in the typical pathology laboratory, but the application of DRAQ5 and eosin do not have a negative effect on later H&E processing as shown in our results, where sections were stained for H&E after D&E staining with no apparent detrimental effects on the resulting H&E appearance. In the case of the clear cell carcinoma (Fig 5), the lack of eosin closely recapitulated the “clear cell” nature of the cells on subsequent H&E, further validating the D&E technique as useful in tumor analysis. Each histology lab has its own method of H&E staining and multiple subtle color variations can be seen across these labs. A benefit to this pseudocoloring process is that the hue of the resulting images can be adjusted to pathologist preference and maintained uniformly across samples. However, the RGB mapping coefficients may be adjusted to recreate color differences in H&E sections that can result from variations in H&E staining intensity and illumination white-balance in brightfield imaging systems. The pseudocolored D&E sections fall along this spectrum and resemble H&E closely enough that any pathologist on initial examination would be comfortable with accurate tissue evaluation.

We have previously demonstrated the ability to diagnose thick tissues using acridine orange and SIM [28, 31]. While the pseudocoloring process can be used with fluorophores other than DRAQ5 and Eosin, DRAQ5’s uniqueness as a DNA-selective, membrane permeant dye with far-red spectral properties provides the potential for it to be paired with many different fluorophores with little spectral interference [32]. DRAQ5 is also highly specific to DNA content in live cells, which stains such as propidium iodide cannot match [33]. The protocol we developed for DRAQ5 allows it to be used in a clinical setting in a relatively short time frame. Staining time and concentration for DRAQ5 is variable in both the literature from the provider’s protocol, and more studies must be completed to characterize its use in fresh, intact tissue [25, 33, 34]. In this work, we held concentration (50 μM) and incubation time (3 minutes) in DRAQ5 constant regardless of tissue thickness. DRAQ5 protocols range in concentration from 1 μM to 500 μM with either immediate viewing or incubation times up to 30 minutes, with the provider protocol stating a 5μM concentration and 10–30 minute incubation [32–35]. To the best of our knowledge, no studies on the performance of DRAQ5 in thick, intact tissues have been completed. Exactly how previous use on cells in culture translates to thick, intact tissue has yet to be described, but our own study shows no marked difference in DRAQ5’s performance on thin, fixed tissue sections compared to imaging the surface of stained, uncut and unfixed biopsies. In comparison, acridine orange is significantly less expensive than DRAQ5 with proven performance on thick tissues. However, acridine orange’s propensity to stain cytoplasmic RNA as well as other structures such as muscle fibers and collagenous stroma limits its utility as a direct analogue to hematoxylin for nuclear-specific staining [20–22]. An in-depth comparison of acridine orange to D&E in terms of contrast mechanism, optimal staining protocols, and cost will be the subject of a future publication.

In this study, the use of D&E as a novel dual stain method is demonstrated as a rapid and efficient means to obtain high quality histology-like images from fresh, unsectioned tissues. This fluorescent stain and processing system creates a digital image that pathologists can quickly review for diagnostic features, similar to an H&E slide, which is ideal as an alternative to standard FSA to preserve tissue content and can easily be paired with standard processing methods. Although we used fluorescence structured illumination microscopy in this work due to its high imaging speed, we have also used confocal microscopy with D&E with excellent results. As this paper was in revision, a study by Giacomelli et al. [36] was published describing a virtual H&E color mapping model which more faithfully captures the physical differences between linear luminous contrast in fluorescence microscopy and non-linear (exponential) absorptive contrast in brightfield microscopy, than the linear mapping model adopted in our work. The use of that algorithm could eliminate the need for the nonlinear intensity adjustments used in our work while maintaining its high dynamic range, and may be a highly useful method for the stain combination and application described here. In summary, fluorescence *ex vivo* microscopy with D&E may thus provide damage-free tissue evaluation and time-efficient turn-around at the point of tissue acquisition in cases where time or tissue conservation is a limiting factor.

## Supporting Information

S1 Fig Comparison of cell nuclei segmentation between D&E (left column) and H&E (right column). The D&E (A) and H&E (B) images from Fig 1L and Fig 1M are shown in the top row. The DRAQ5 channel corresponding to cell nuclei is shown in (C), whereas the cell nuclei segmented from the H&E image using the standard ImageJ color deconvolution plugin is shown in (D). Although the H&E image was collected at a higher magnification and resolution (20X, 1 μm resolution) than the DRAQ5 image (10X, 1.9 μm resolution), the close morphological correspondence between the DRAQ5-labeled nuclei and the nuclei segmented from the H&E image are apparent. The use of DRAQ5 may in fact offer a more accurate segmentation of nuclei in histologic images than the use of color deconvolution on H&E images, as shown in the contrast-enhanced versions in (E) and (F). The use of color deconvolution to segment areas stained by hematoxylin results in extraction of image areas not associated with cell nuclei (F), whereas the DRAQ5 channel is highly specific to the cell nuclei (E).
(TIF)

S2 Fig Quantitative comparison of nuclear area between DRAQ5 channel and segmented H&E. Cell nuclei in a single prostatic gland were manually outlined in ImageJ in the segmented H&E image (A) and the DRAQ5 channel of the D&E image (B). Nuclei are observed to be highly similar in size and shape and measured nuclear areas (C) were comparable between the two methods–the slightly higher areas in the DRAQ5 channel may be attributed to the larger pixel size and lower optical resolution of the D&E images compared to the H&E images.
(TIF)
