# Supplementary figures and images for: DRAQ5 and Eosin (‘D&E’) as an Analog to Hematoxylin and Eosin for Rapid Fluorescence Histology of Fresh Tissues
Source: PLoS One. 2016 Oct 27;11(10):e0165530. doi: 10.1371/journal.pone.0165530 (PMC5082869; doi:10.1371/journal.pone.0165530)

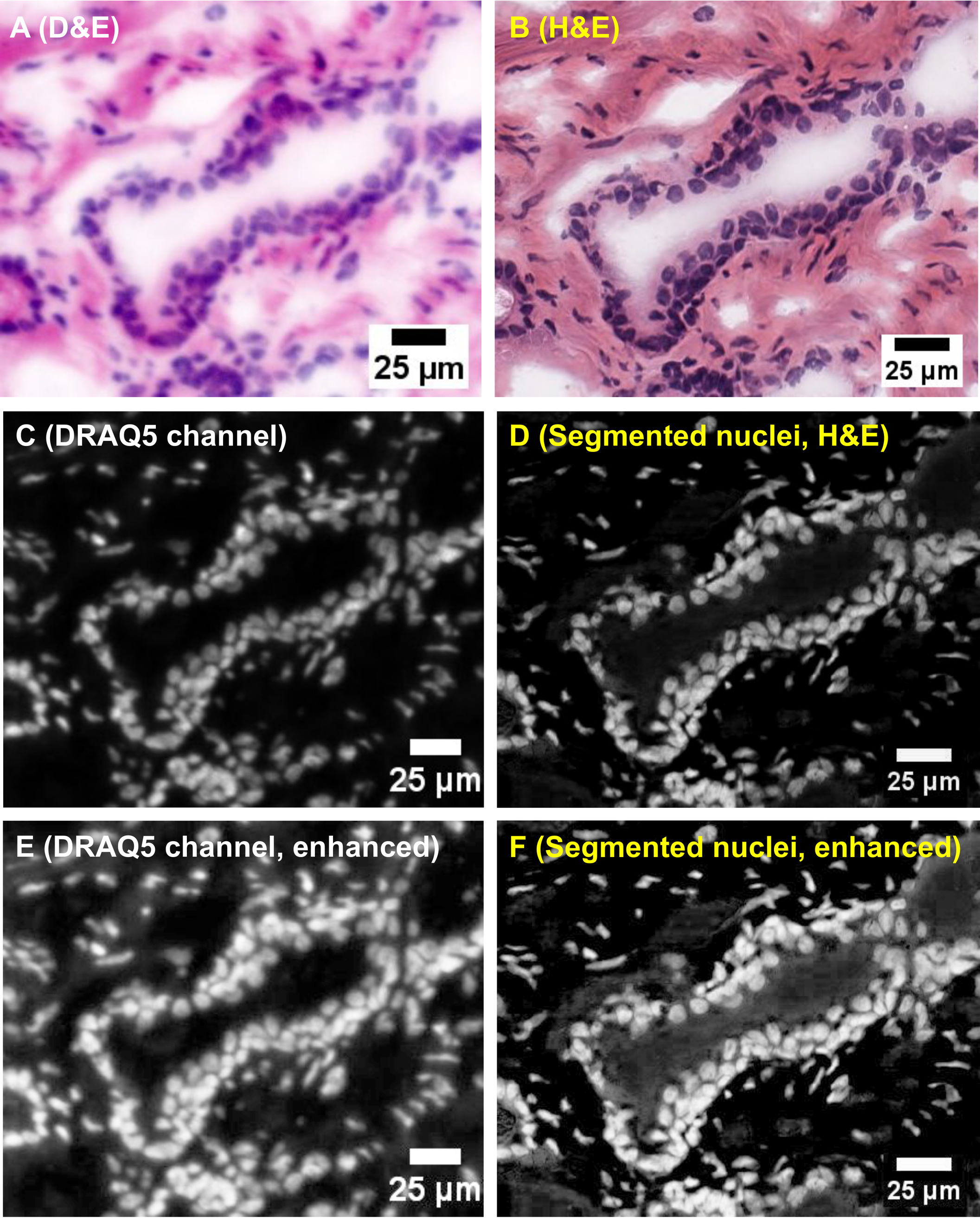

Supplement: S1 Fig — The D&E (A) and H&E (B) images from Fig 1L and Fig 1M are shown in the top row. The DRAQ5 channel corresponding to cell nuclei is shown in (C), whereas the cell nuclei segmented from the H&E image using the standard ImageJ color deconvolution plugin is shown in (D). Although the H&E image was collected at a higher magnification and resolution (20X, 1 μm resolution) than the DRAQ5 image (10X, 1.9 μm resolution), the close morphological correspondence between the DRAQ5-labeled nuclei and the nuclei segmented from the H&E image are apparent. The use of DRAQ5 may in fact offer a more accurate segmentation of nuclei in histologic images than the use of color deconvolution on H&E images, as shown in the contrast-enhanced versions in (E) and (F). The use of color deconvolution to segment areas stained by hematoxylin results in extraction of image areas not associated with cell nuclei (F), whereas the DRAQ5 channel is highly specific to the cell nuclei (E). (TIF) [file pone.0165530.s001.tif]

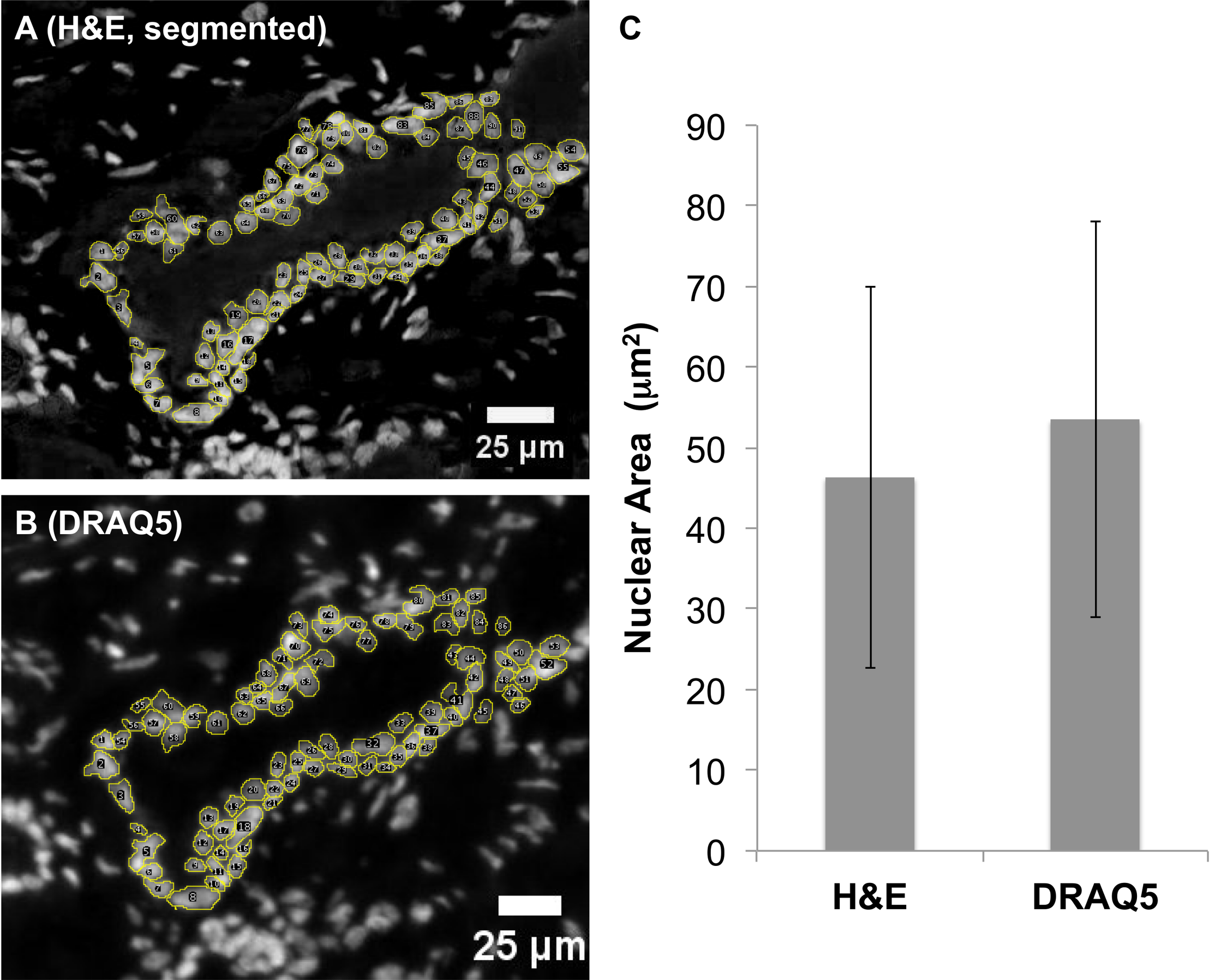

Supplement: S2 Fig — Cell nuclei in a single prostatic gland were manually outlined in ImageJ in the segmented H&E image (A) and the DRAQ5 channel of the D&E image (B). Nuclei are observed to be highly similar in size and shape and measured nuclear areas (C) were comparable between the two methods–the slightly higher areas in the DRAQ5 channel may be attributed to the larger pixel size and lower optical resolution of the D&E images compared to the H&E images. (TIF) [file pone.0165530.s002.tif]
